# Chaperonin of Group I: Oligomeric Spectrum and Biochemical and Biological Implications

**DOI:** 10.3389/fmolb.2017.00099

**Published:** 2018-01-25

**Authors:** Silvia Vilasi, Donatella Bulone, Celeste Caruso Bavisotto, Claudia Campanella, Antonella Marino Gammazza, Pier L. San Biagio, Francesco Cappello, Everly Conway de Macario, Alberto J. L. Macario

**Affiliations:** ^1^Institute of Biophysics, National Research Council, Palermo, Italy; ^2^Section of Human Anatomy, Department of Experimental Biomedicine and Clinical Neuroscience (BIONEC), University of Palermo, Palermo, Italy; ^3^Euro-Mediterranean Institute of Science and Technology (IEMEST), Palermo, Italy; ^4^Department of Microbiology and Immunology, School of Medicine, University of Maryland at Baltimore, and Institute of Marine and Environmental Technology (IMET), Columbus Center, Baltimore, MD, United States

**Keywords:** Hsp60, GroEL, monomer, heptamer, tetradecamer, post-translation modification, chaperonopathies, non-canonical locales

## Abstract

Chaperonins play various physiological roles and can also be pathogenic. Elucidation of their structure, e.g., oligomeric status and post-translational modifications (PTM), is necessary to understand their functions and mechanisms of action in health and disease. Group I chaperonins form tetradecamers with two stacked heptameric rings. The tetradecamer is considered the typical functional complex for folding of client polypeptides. However, other forms such as the monomer and oligomers with smaller number of subunits than the classical tetradecamer, also occur in cells. The properties and functions of the monomer and oligomers, and their roles in chaperonin-associated diseases are still incompletely understood. Chaperonin I in eukaryotes occurs in various locations, not just the mitochondrion, which is its canonical place of residence and function. Eukaryotic Chaperonin I, namely Hsp60 (designated HSP60 or HSPD1 in humans) has, indeed, been found in the cytosol; the plasma-cell membrane; on the outer surface of cells; in the intercellular space; in biological liquids such as lymph, blood, and cerebrospinal fluid; and in secretions, for instance saliva and urine. Hsp60 has also been found in cell-derived vesicles such as exosomes. The functions of Hsp60 in all these non-canonical locales are still poorly characterized and one of the questions not yet answered is in what form, i.e., monomer or oligomer, is the chaperonin present in these non-canonical locations. In view of the steady increase in interest on chaperonopathies over the last several years, we have studied human HSP60 to determine its role in various diseases, its locations in cells and tissues and migrations in the body, and its post-translational modifications that might have an impact on its location and function. We also carried out experiments to characterize the oligomeric status of extramitochondrial of HSP60 in solution. Here, we provide an overview of our results, focusing on the oligomeric equilibrium and stability of the various forms of HSP60 in comparison with GroEL. We also discuss post-translational modifications associated with anti-cancer drugs to indicate the potential of Hsp60 in Medicine, as a biomarker and etiopathogenic factor.

Research on molecular chaperones is steadily increasing not only because they are key elements in cellular and organismal normal physiology but also because, if abnormal, they can become etiopathogenic and contribute to the development of diseases, the chaperonopathies. These are pathologic conditions in which chaperones abnormal in composition-structure (e.g., mutations or post-translational modifications), quantitative levels, location in the cell or outside it, or function (loss or excess of it, gain of new function) play an etiopathogenic role, either primary or auxiliary. Chaperonins of Group I and II have indeed been identified as pathogenic factors in a number of conditions. For example, chaperonins of Group I, the object of this review, can cause serious diseases (a subgroup of chaperonopathies that may be called chaperoninopathies) if mutated at critical sites (Bross et al., 2008; Bross and Fernandez-Guerra, 2016), or can contribute to the initiation and/or progression of certain types of cancer, and chronic and autoimmune disorders (Macario et al., 2013; Cappello et al., 2014; Wick et al., 2014; Wick, 2016; Rahman et al., 2017; van Eden et al., 2017; Calderwood, 2018; Pockley and Henderson, 2018).

While the clinical and pathological manifestations of many chaperonopathies are reasonably well-characterized, the pathogenic mechanisms underpinning the lesions observed in tissues and organs of patients are still poorly understood. For example, little is known on the impact of point-mutations on the chaperone molecule's intrinsic properties (e.g., stability in the face of stressors, ability to interact with the required partners to exercise chaperoning functions, flexibility, and oligomeric organization in solution) and chaperoning and non-chaperoning (moonlighting) functions, inside, or outside the cell. It is, therefore, imperative to elucidate the properties of the chaperone molecules under physiologic conditions and in situations resembling those occurring in patients as a required preliminary step to improving diagnosis, treatment, and prevention of chaperonopathies. Here, we briefly discuss some results pertaining to the Group I chaperonins Hsp60 and GroEL, obtained in a variety of laboratories, including ours.

The best characterized chaperonin of Group I, GroEL, forms tetradecamers with two stacked heptameric rings (Horwich et al., 2007). The tetradecamer is the typical functional complex that carries out the folding of client polypeptides. However, in mammalian cells, other forms of the Group I chaperonin, such as the monomer and oligomers with smaller number of subunits than the classical tetradecamer, are known to occur. A single ring seems to be sufficient for productive protein folding *in vivo* (Nielsen and Cowan, 1998), and the requirement of double rings football-shaped intermediates is still under debate (Viitanen et al., 1992). The properties and functions of the monomer and oligomers are also incompletely understood. Likewise, the impact of oligomeric organization on Hsp60 loss of function of some pathological situations has not yet been elucidated, despite the fact that this is a particularly interesting issue since destabilization of the mitochondrial HSP60 oligomer characterizes the MitCHAP-60 disease, a severe neurodegenerative condition associated to the Asp3Gly mutation in HSP60 (Parnas et al., 2009).

Chaperonin I in eukaryotes occurs in various locations, not just the mitochondrion, which is its canonical place of residence and function. Eukaryotic Chaperonin I, namely Hsp60 (designated HSP60 or HSPD1 in humans) has, indeed, been found in the cytosol; the plasma-cell membrane; on the outer surface of cells; in the intercellular space; in biological liquids such as lymph, blood, and cerebrospinal fluid; and in secretions, for instance saliva and urine (Cechetto et al., 2000; Cappello et al., 2008; Macario et al., 2013; Calderwood, 2018; Pockley and Henderson, 2018; more references can be found at http://hsp60.com/localization/). Extra-mitochondrial functions of HSP60 have also been observed in yeast (Kalderon et al., 2015). Hsp60 has also been found in cell-derived vesicles such as exosomes (Merendino et al., 2010; Campanella et al., 2012). The functions of Hsp60 in all these non-canonical locales are still poorly characterized and one of the questions not yet answered is in what form, i.e., monomer or oligomer, is the chaperonin present in these non-canonical locales.

Over the last several years we have studied human HSP60 in several systems to determine its location in cells and tissues, its migrations in the body, and its post-translational modifications that might have an impact on its location and function. We have also carried out experiments to elucidate the oligomeric status of human extramitochondrial HSP60 in solution and we have investigated its role in various diseases. Our observations are discussed in this brief review along with pertinent data from other laboratories.

The nomenclature of heat shock proteins and molecular chaperones is rather confusing and to facilitate the reading of this review we will use the following terms: chaperoning system is the complete set of chaperones, co-chaperones, and chaperone co-factors, and other closely interacting receptors and molecules of an organism. A chaperoning team is the functional complex formed by two or more identical or closely related subunits, e.g., the tetradecamer characteristic of GroEL and HSPD1. Teams participate in networks which consist of two or more interacting teams, e.g., Hsp70-prefoldin-Hsp90. We will use the terms HSP60 or HSPD1 for the human chaperonin of Group I, but in referring to this chaperonin in general, without specifying the species we will use Hsp60.

## The human HSP60 and associated diseases

The realization that abnormal chaperones can cause disease opened new avenues to study chaperones and provided incentive to re-evaluate these molecules by medical researchers and practitioners (Macario and Conway de Macario, 2005). A case in point is human HSP60 because it was discovered that mutations in it caused specific diseases (Bross and Fernandez-Guerra, 2016). Noteworthy is that the ability of the mutant chaperonin to form oligomers was affected (Parnas et al., 2009). In addition, a missense mutation, Leu73Phe, of HSP10, the co-chaperone of HSP60, was recently reported to be associated to a severe neurological and developmental disorder (Bie et al., 2016). The detailed molecular mechanism involving the mutant HSP10 that causes the lesions observed has not yet been fully elucidated. However, studies with the purified mutant protein *in vitro* demonstrated that it has poor thermal stability, and its refolding ability and resistance to proteolysis are very much impaired. The availability of this mutant offers a unique opportunity to study the impact of the mutation on the interactions between HSP10 and HSP60 and, thereby, learn about the intrinsic mechanism of formation of the complete HSP60/HSP10 complex.

Hsp60 is classically considered a mitochondrial protein with its coding gene residing in the cell nucleus. This implies that after being synthesized in the cytosol, the Hsp60 protein must migrate into mitochondria. Therefore, one may predict that there are at least two intracellular populations of Hsp60, one present in the cytosol and the other inside the organelle. In some pathological conditions, this equilibrium can be altered, with abnormal accumulation of Hsp60 in the cytosol. In addition, as mentioned above, Hsp60 has been found in other locations beyond the mitochondrion. It is generally believed that the intramitochondrial chaperoning function of Hsp60 requires the formation of tetradecamers but little is known about the structure of the chaperonin in other locations. In this brief review we examine some of the properties of the spectrum of Hsp60 forms that have been identified, monomers, heptamers, and tetradecamers. We discuss the possibility that the human HSP60 chaperonin may also function as a single ring and may form symmetrical football-shaped intermediates, similarly to GroEL.

## The human HSP60 gene (*HSPD1*)

The human mitochondrial chaperonin HSP60 is encoded in the gene *HSPD1* (also written *hspd1*) which is localized in chromosome 2 head to head with the gene that codes for the HSP10 co-chaperonin, designated *HSP10* (also written *cpn60* or *HSPE1*), and the two genes are separated by a bidirectional promoter (Hansen et al., 2003).

**Figure 1:**
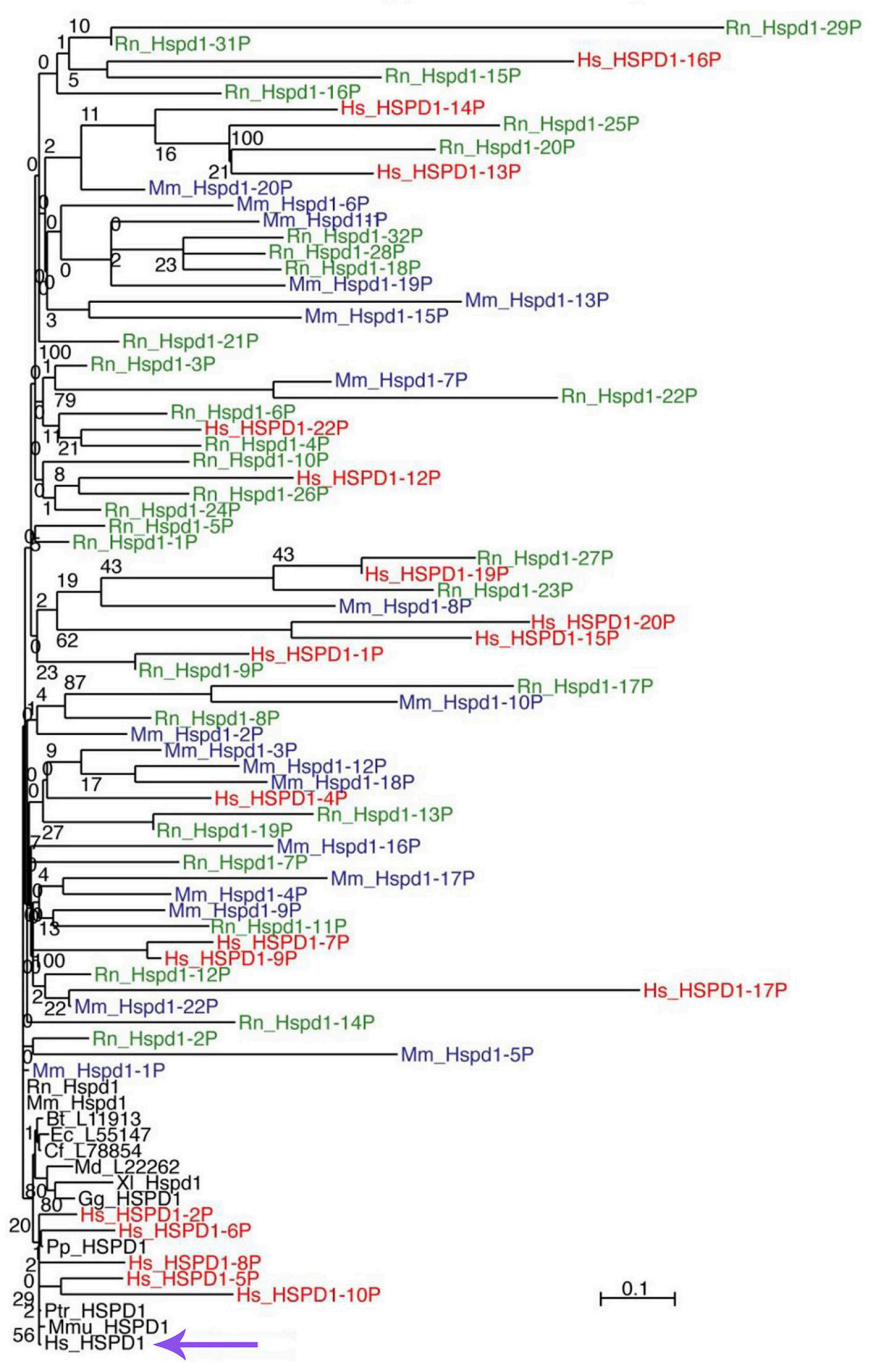
ML tree of mammalian Hsp60 (Cpn60) sequences, including genes (black font) and pseudogenes from human (red font), mouse (blue font), and rat (green font). The scale bar represents the indicated number of substitutions per position for a unit branch length. The species tested are not all included in this representation of the tree to avoid overcrowding. In the following list of abbreviations of the names of the species tested, those shown in the figure are in bold face: **Bt**, ***Bos taurus*** (cow); **Cf**, ***Canis lupus familiaris*** (dog); Dn, *Dasypus novemcinctus* (nine-banded armadillo); Dr, *Danio rerio* (zebrafish); **Ec**, ***Equus caballus*** (horse); Ga, *Gasterosteus aculeatus* (stickleback, fish); **Gg**, ***Gallus gallus domesticus*** (chicken); **Hs**, ***Homo*** ***sapiens*** (human); La, *Loxodonta africana* (african bush elephant); **Md**, ***Monodelphis*** ***domestica*** (south american gray short-tailed opossum, marsupial); **Mm**, ***Mus musculus*** (mouse); **Mmu**, ***Macaca mulatta*** (rhesus monkey); Mmur, *Microcebus murinus* (gray mouse lemur); Oa, *Ornithorhynchus anatinus* (platypus); Ol, *Oryzias latipes* (the medaka or japanese killifish); **Pp**, ***Pongo pygmaeus*** (northwest bornean orangutan); **Ptr**, ***Pan troglodytes*** (chimpanzee); **Rn**, ***Rattus norvegicus*** (rat); Tn, *Tetraodon nigroviridis* (spotted green pufferfish); Tr, *Takifugu rubripes* (japanese pufferfish); **Xl**, ***Xenopus laevis*** (african clawed frog, amphibian); Xt, *Xenopus tropicalis* (western clawed frog, amphibian). The violet arrow at the bottom indicates the human HSPD1.

**Figure 2:**
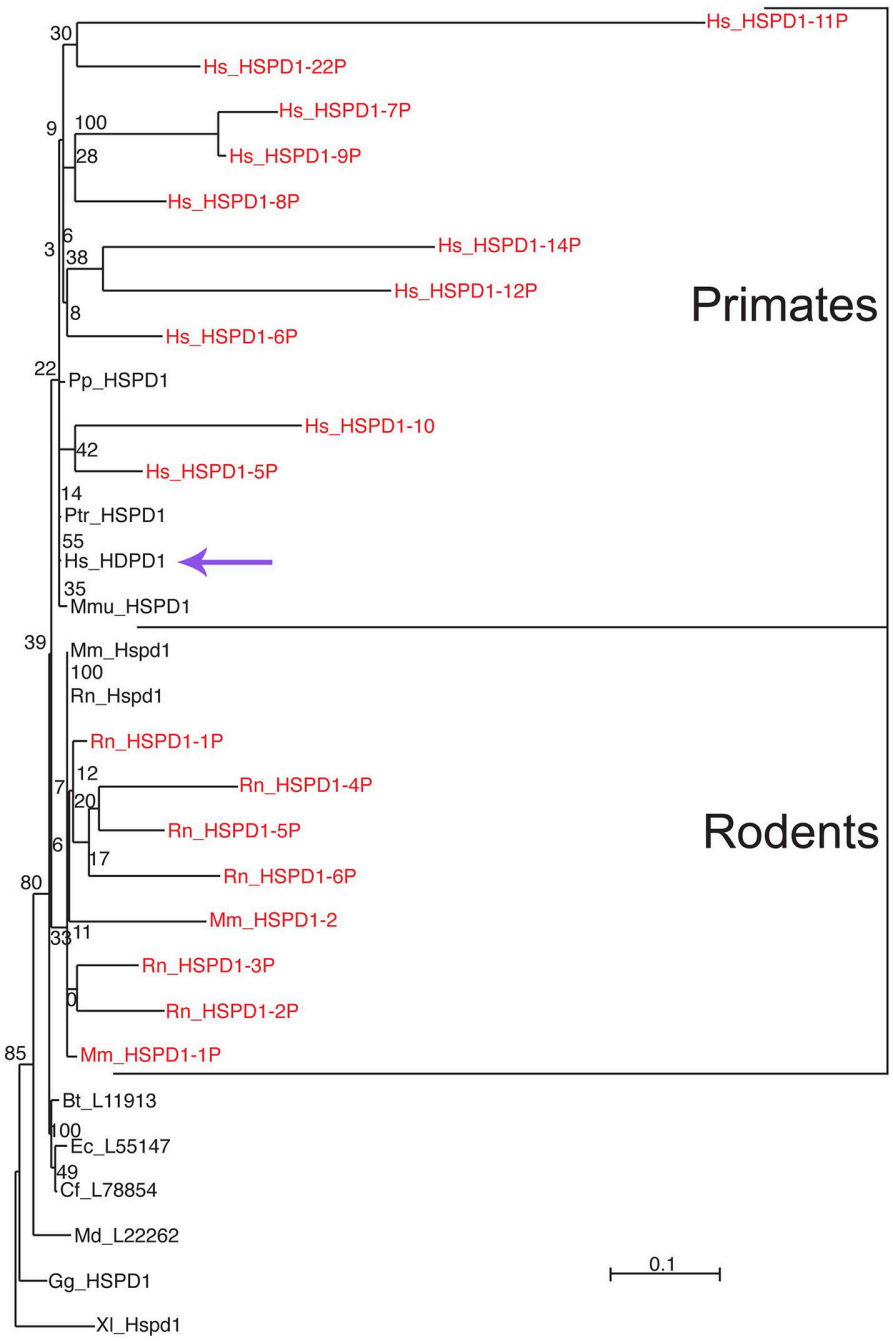
Differential clustering of human, mouse, and rat Hsp60-related pseudogenes. Pseudogenes are in red font. Human pseudogenes are clustered with primate Hsp60 (Cpn60) sequences whereas mouse and rat pseudogenes are clustered with rodent counterparts, indicating independent evolution of these pseudogenes in these species. For species abbreviations see legend for Figure 1. The scale bar represents the indicated number of substitutions per position for a unit branch length. The violet arrow indicates the human HSPD1. Source: Mukherjee et al. (2010) (Original publisher BioMed Central: BMC Evol Biol.).

Only one *HSPD1* gene and 22 *HSPD1* pseudogenes were found in the human genome (Mukherjee et al., 2010). Figure 1 shows a Maximum Likelihood (ML) tree of mammalian Hsp60 (Cpn60) proteins, including genes and pseudogenes from human, mouse, and rat. Interestingly, ML trees built with translations of only the most conserved pseudogenes showed consistent association of the human pseudogenes with Hsp60 from primates, whereas pseudogenes from mouse and rat all associated with murid Hsp60 sequences, indicating their relatively recent origin, Figure 2. The human HSP60 pseudogenes are distributed over several chromosomes (1, 3, 4, 5, 6, 8, 10, 11, 12, 13, 20, and 21) but none is present on chromosome 2, in which the *HSPD1* gene is located.

While there is considerable information on the genomics of *HSPD1* available from studies directly on the human genome, knowledge of the structure and properties of the human protein has recently advanced based on the crystallization of the human mitochondrial HSP60 (Nisemblat et al., 2014, 2015), and is still mostly grounded to a considerable extent on studies of prokaryotic, especially bacterial GroEL.

## GroEL

The study of Group I chaperonins was stimulated by seminal reports that appeared in the late 1970's and the 1980's. For example, a gene was identified in which certain mutations directly correlated to impairment of bacteriophage λ growth, and the gene product was found to be a 60 kDa protein, which was later named GroEL (Georgopoulos and Hohn, 1978). A similar protein was identified in chloroplasts, associated with Rubisco with an amino-acid sequence over 40% similar to GroEL (Ellis and van der Vies, 1988). This protein was characterized by “assisting” functions, which inspired the name “molecular chaperone” (Ellis et al., 1989).

In another work, it was established that a mitochondrial protein had the ability to assist protein folding and that increased in response to heat shock (McMullin and Hallberg, 1988). This protein formed two stacked heptameric rings (Ostermann et al., 1989) and was found to have the ability of self-assembling (Cheng et al., 1990). It was also proposed that these 60 kDa proteins present in chloroplasts, mitochondria, and bacteria such as *Escherichia coli*, and whose genes were heat-inducible should be named chaperonins (Hemmingsen et al., 1988). Subsequent work in many laboratories (Saibil et al., 2013; Nisemblat et al., 2015; Okamoto et al., 2015; Weiss et al., 2016; Chen et al., 2017; Illingworth et al., 2017; Ishii, 2017; Liu et al., 2017; Roh et al., 2017) unveiled the structure and chaperoning cycle of the Hsp60 chaperonins and revealed various features that are shared by these proteins, for instance the formation of rings and more complex structures made of two stacked rings with a central cavity or cage, which was found to be essential in client polypeptide folding.

The typical GroEL complex is constituted of two identical rings with seven identical subunits each, so the complete team is a homo-tetradecamer with a central cavity or cage (Hohn et al., 1979). The GroEL subunit has three domains, apical, intermediate, and equatorial. While GroEL is typically present in bacteria, similar and evolutionarily-related structures also occur in some archaea (e.g., *Methanosarcina mazei*; Conway de Macario et al., 2003), in the mitochondria of eukaryotes, for instance *Neurospora crassa* (Hutchinson et al., 1989) and humans (Hansen et al., 2003; Mukherjee et al., 2010), and in the chloroplast of plants (Dickson et al., 2000).

**Figure 3:**
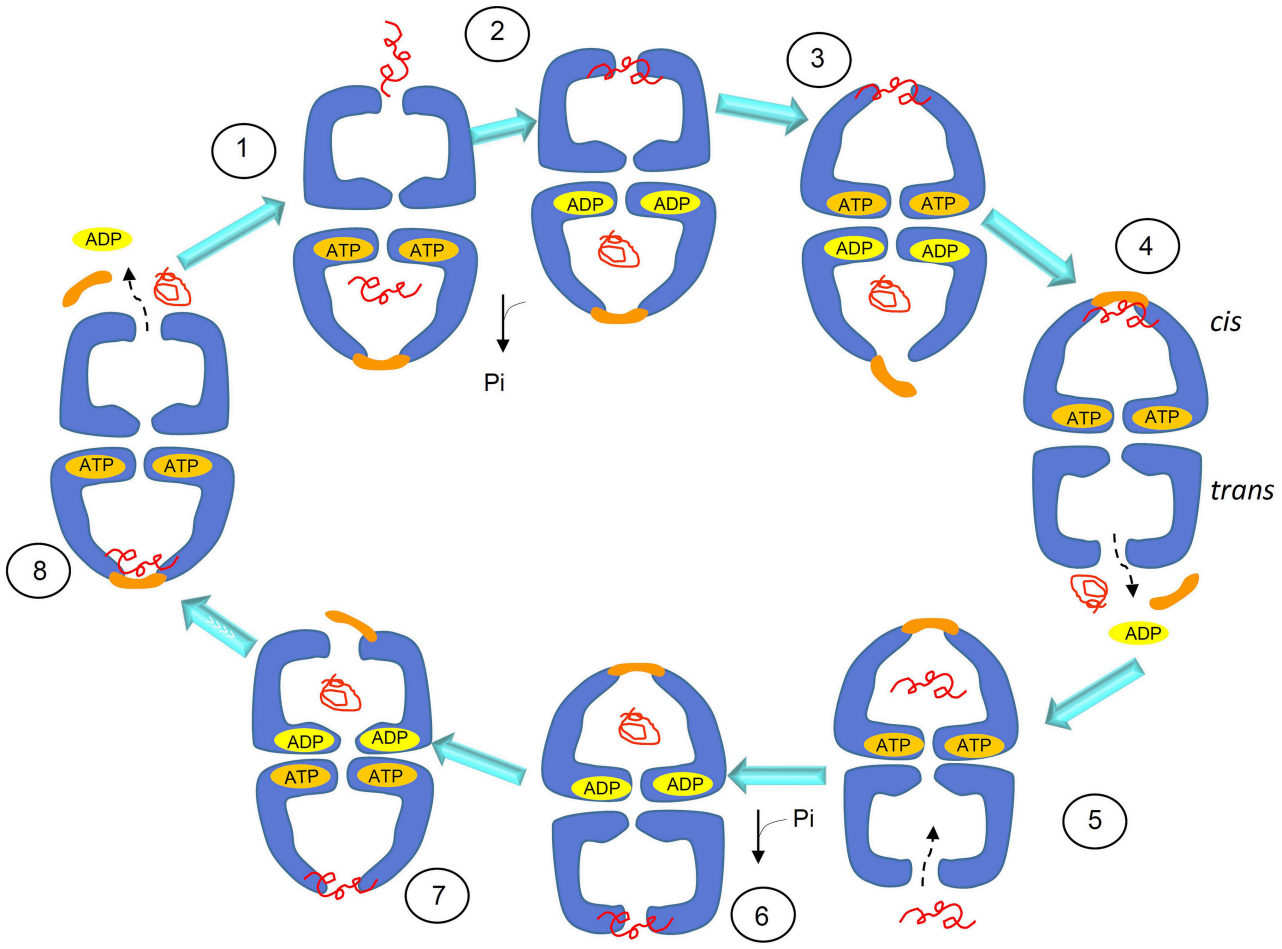
Schematics of the substrate-folding cycle of GroEL. This model of the cycle involves asymmetric, bullet-shaped intermediates and proceeds as follows: when the substrate (the client peptide in need of assistance for folding) binds to the apical domain of one of the two rings, the bound ring rearranges to form a cis complex with GroES, allowing substrate encapsulation inside the cage and folding with energy from ATP hydrolysis. GroES binding to the *cis* ring can occur only when ADP and the folded protein are released to the outside from the cage of the *trans* ring. Therefore, the cycle intermediate is an asymetric bullet-shaped oligomeric chaperoning complex. Adapted with permission from Macmillan Publishers Ltd. Horwich (2011).

The chaperoning function of GroEL tetradecamers requires allosteric changes and communication between the team members, i.e., subunits and rings (Goloubinoff et al., 1989). The chaperoning mechanism, according to this model, proceeds through a series of coordinated steps and is initiated by the binding of the client polypeptide (substrate) to the apical domain via hydrophobic residues (Horwich, 2011). Then, upon binding of ATP to the equatorial domain the apical and intermediate domains rearrange allowing transition of the complex from *trans* to *cis* conformation, thus the central cavity encapsulates the substrate (Figure 3). The other member of the chaperoning complex, the homo-heptameric GroES, binds the same apical hydrophobic residues, which leads to the release of the substrate into the cage for folding, using energy provided by ATP hydrolysis (Figure 3). ATP hydrolysis in the *cis* ring is followed by binding of ATP to the *trans* ring, which causes dissociation of the *cis* complex, thereby releasing the folded substrate, ADP, and GroES. In this model, the GroES heptamer can bind a ring only after its release from the other, so that the entire chaperoning complex has an asymmetric bullet-shaped structure (Figure 3). Therefore, this model of GroEL function involves two GroEL heptamers and one GroES heptamer, assembled in an asymmetric, bullet-shaped GroEL-GroES complex. This model, widely accepted for many years, is currently challenged by recent single-molecule studies that revealed the presence, during the GroEL cycle, of symmetric, football-shaped intermediates of the GroEL tetradecamer with a GroES heptamer on each end (Taguchi, 2015). These football-shaped structures were stabilized under specific experimental conditions and then crystallized and solved (Fei et al., 2014; Koike-Takeshita et al., 2014). It was found that, due to the structural features of the interface, the negative cooperativity between rings in the football-shaped complex mainly involving two D helices, is reduced as compared with the cooperativity between the rings of the bullet-shaped complex. A GroES heptamer binding in one ring is not dependent on a GroES heptamer being released from the other, and substrate folding can occur at both GroEL rings simultaneously, thus causing the formation of symmetric complexes. The presence of the symmetric complexes is not incompatible with bullet-shaped structures as they can coexist, but, as revealed by Fluorescence Resonance Energy Transfer (FRET), the presence of substrate is crucial for shifting the equilibrium toward symmetric intermediates (Sameshima et al., 2010). This role of the substrate influencing the GroEL oligomeric structural organization and function is in line with results obtained by multi-scale simulations (Coluzza et al., 2008).

Similarly to what happens for the chaperonin from the bacterium *Thermus thermophilus* (Ishii et al., 1995), in the presence of K^+^ and Mg-ATP, the symmetric football-shaped complex undergoes a dynamic transition consisting of equatorial split, which leads to the formation of single rings (Koike-Takeshita et al., 2014). Although the functional role of the split is not yet fully understood, this observation could be relevant to interpret some results obtained with mammalian, e.g., human mitochondrial HSP60, as discussed later in this review.

## From bacteria to mitochondria

In contrast to the abundance of studies on the bacterial Hsp60, research on the human counterpart had a slow start and took off when the amino-acid sequence of the human protein was found similar to those of bacterial and plant chaperonins. By means of immunofluorescence and biochemical fractionation techniques, it was shown that mammalian Hsp60 is primarily present in the mitochondrial matrix (Gupta and Austin, 1987). Despite the high degree of sequence similarity to GroEL, human HSP60 has some peculiar features in oligomer organization. By Gel Filtration and Sucrose Density Gradient Centrifugation, it was found to occur as a homo-oligomer of seven subunits of ~440,000 Mr (Jindal et al., 1989). Single active rings were also found in other mammalian Hsp60, like that from Chinese hamster ovary (CHO) that shares a high sequence similarity with the human counterpart (Picketts et al., 1989).

**Figure 4:**
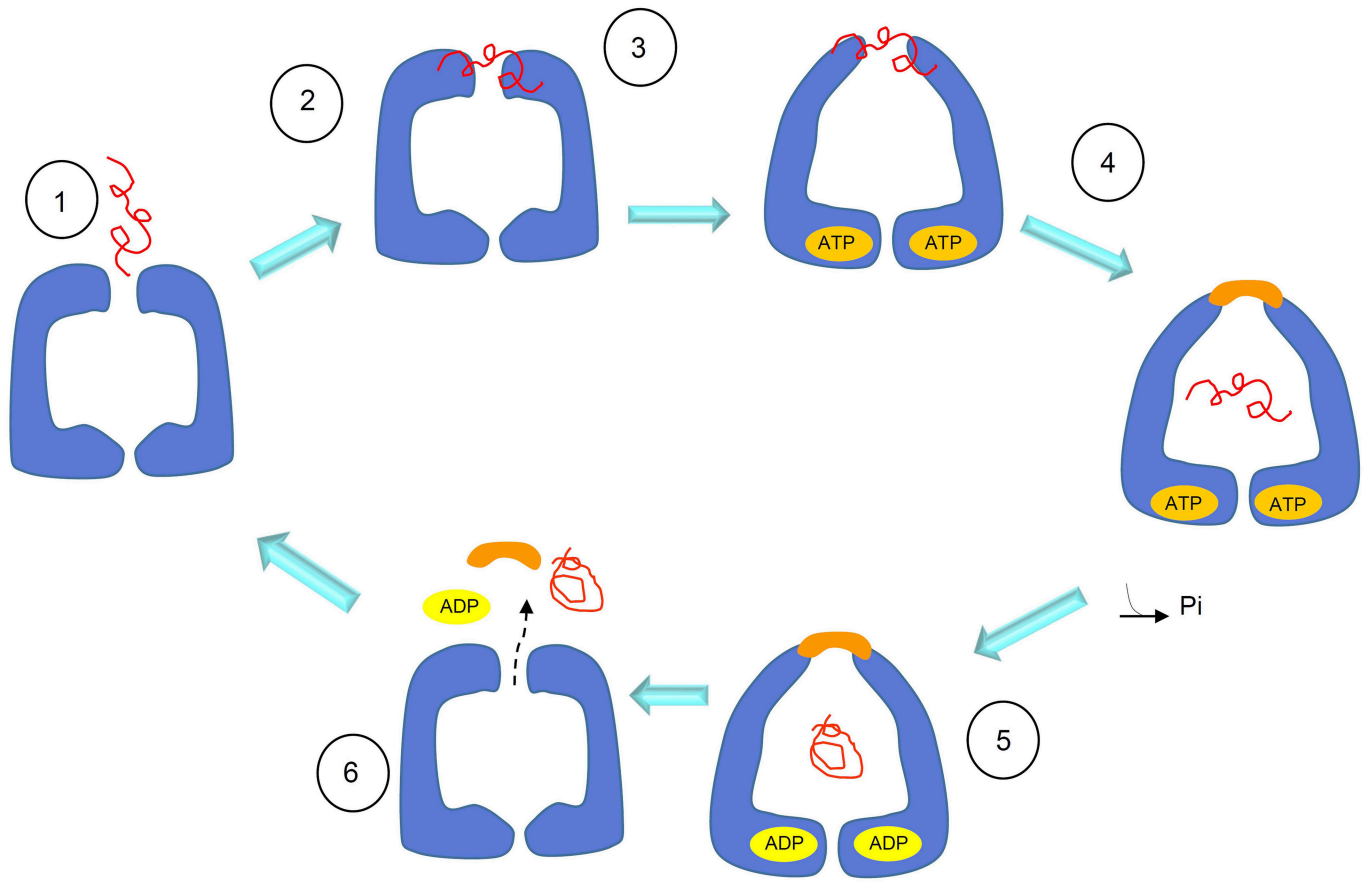
Schematics of the substrate-folding cycle of GroEL single rings. According to this proposed mechanism, when the substrate binds to the apical domain of the single ring, the latter rearranges to form a complex with the Hsp10 heptamer. This allows substrate encapsulation and folding with energy from ATP hydrolysis. In contrast to what happens with the full, bullet-shaped complex described in Figure 3, the interaction between the single Hsp60 ring with the Hsp10 heptamer in the presence of ADP becomes so weak that the ring opens and both, the Hsp10 heptamer and the client-protein are released. Adapted with permission from Macmillan Publishers Ltd. Horwich (2011).

When a protocol to purify recombinant Hsp60 without a His-tag became available (Viitanen et al., 1992), extensive studies were performed to understand the structure and oligomeric organization of the mammalian mitochondrial chaperonin. Moreover, it became possible to cleave the N-terminal mitochondrial targeting signal (MTS) present in the eukaryotic Hsp60 and obtain the mature Hsp60 as it occurs inside mitochondria, designated mtHsp60. The protein was mainly obtained as monomers and heptamers. This seems to be a general result: in the absence of ATP and mtHsp10 (mitochondrial Hsp10, or Cpn10), mtHsp60 occurs primarily as a single ring with a mass of 440 kDa (Levy-Rimler et al., 2001). Tetradecamers would form only upon dissociation of the initial oligomers in the presence of ATP at low temperature, followed by incubation at 30°C with mtHsp10 and ATP (Viitanen et al., 1992). Surprisingly, the mammalian mtHsp60 as single toroidal ring was found to be able, in the presence of Mg-ATP and mtHsp10, of facilitating folding of non-native ribulase-P2 carboxylase, forming with it a stable complex. However, in these experiments, a two-ring complex seemed to be an obligatory participant in productive substrate folding (Viitanen et al., 1992). The formation of single rings was also detected, in the absence of substrate, in studies on human mtHSP60 (Nielsen and Cowan, 1998). By electron microscopy (EM), single rings appeared not only from wild type HSP60, but also as chimeric complexes composed of equatorial HSP60 and apical GroEL. Instead, wild type GroEL and chimeric complexes formed by HSP60 apical domain and GroEL equatorial domain appeared as tetradecamers (Nielsen and Cowan, 1998). These observations showed that the ability to form single or double rings is confined to the equatorial part of the protein and in particular, as observed for the mutant GroEL_SR1_, crucial are the residues R452, E464, S463, and V464, that, when mutated into alanine, cause GroEL to form single rings, too. However, contrary to wild type Hsp60, GroEL_SR1_ is unable to release client proteins, with the exception of rhodanese. Instead, Hsp60_SR1_, a mutant with mutations at the same positions of mutated residues of GroEL_SR1_, and considered to be unable of forming tetradecamers, refolds and releases client proteins. It can, therefore, be inferred that the formation of tetradecamer intermediates, as discussed earlier (Viitanen et al., 1992), is not obligatory for productive folding of substrates, and that an alternative pathway may exist (Figure 4) (Nielsen and Cowan, 1998; Weiss et al., 2016).

More recently, a mutant (E321K) of the human mtHSP60 was crystallized and its structure solved (Weiss et al., 2016). The mutation confers high stability to the open conformation of mtHSP60 so to generate a very stable complex between mtHSP60 and mtHSP10, and with a molecular symmetry that facilitated its crystallization. Thus, the structure of this complex was revealed by X-ray Diffraction Analysis, showing the existence of a football-shaped double-ring oligomer that, although similar to that found for GroEL differs from it in various features (Nisemblat et al., 2014). Similarly to GroEL, the N-terminal and C-terminal regions of HSP60 are located in the equatorial domain of the protein that is involved in the inter-ring interface of the oligomeric chaperonin. The rings can both simultaneously bind ATP and one of the seven monomers in each ring is somewhat different from the others so to create an internal asymmetry. In the football-shape model, the rings independently bind substrate, ATP, and mtHsp10 and, only after that, they join together before encapsulating and refolding the substrate. The mutant mtHsp60E321K, even if not able to release the folded substrate, can encapsulate and refold client proteins such as the Enhanced Green Fluorescent Protein (EGFP). Therefore, it cannot be excluded that the symmetric, football-shaped intermediate is present also in the chaperoning cycle of the wild type mtHsp60.

In conclusion, it can be hypothesized that different functional mechanisms, involving various types of oligomers, may occur with Hsp60 single rings (Nielsen and Cowan, 1998; Weiss et al., 2016). It is likely that the chaperonin may follow a given pathway depending on substrate type and on micro-environmental conditions such as those occurring under physiological and stress situations.

## Hsp60 outside mitochondria

In eukaryotic cells Hsp60 is typically located inside mitochondria, which presumably evolved from prokaryotes via endosymbiosis (Gupta, 1995). mtHsp60 is encoded in a nuclear gene and is synthesized in the cytosol with an MTS. This N-terminal sequence forms a positively charged amphiphilic α-helix, which is essential for mitochondrial import. The post-translational import of Hsp60 into mitochondria is, like for other cytosolic proteins that translocate into the organelle, a very intricate process that involves protein translocation complexes such as TOM in the outer membrane and TIM in the inner membrane. The import mechanism is regulated by the membrane potential ΔΨ that, when dissipated by potassium ionophores, inhibits the maturation of Hsp60 (Gupta and Austin, 1987).

The sequence of MTS in the human HSP60 was deduced from the cDNA entire gene sequence compared with that of the matured protein, in which the N-terminal MTS was missing. The MTS consists of 26 amino acids: 5′-Met-Leu-Arg-Leu-Pro-Thr-Val-Phe-Arg-Gln-Met-Arg-Pro-Val-Ser-Arg-Val-Leu-Ala-Pro-His-Leu-Thr-Arg-Ala-Tyr-3′. Based on studies on GroEL structure (Clare et al., 2012), it can be expected that the MTS fits inside the central cavity of HSP60 oligomers. Structural details on naïve HSP60 based on SAXS and Molecular Dynamics studies can be found in a relatively recent report (Spinello et al., 2015).

When the Hsp60 N-terminus is analyzed by PONDR VLXT, a neural networks disorder predictor, it appears that in the absence of MTS, residues adjacent to it undergo an order-disorder transition, probably related to the functional role that the chaperonin plays in mitochondria (Ricci et al., 2017).

**Figure 5:**
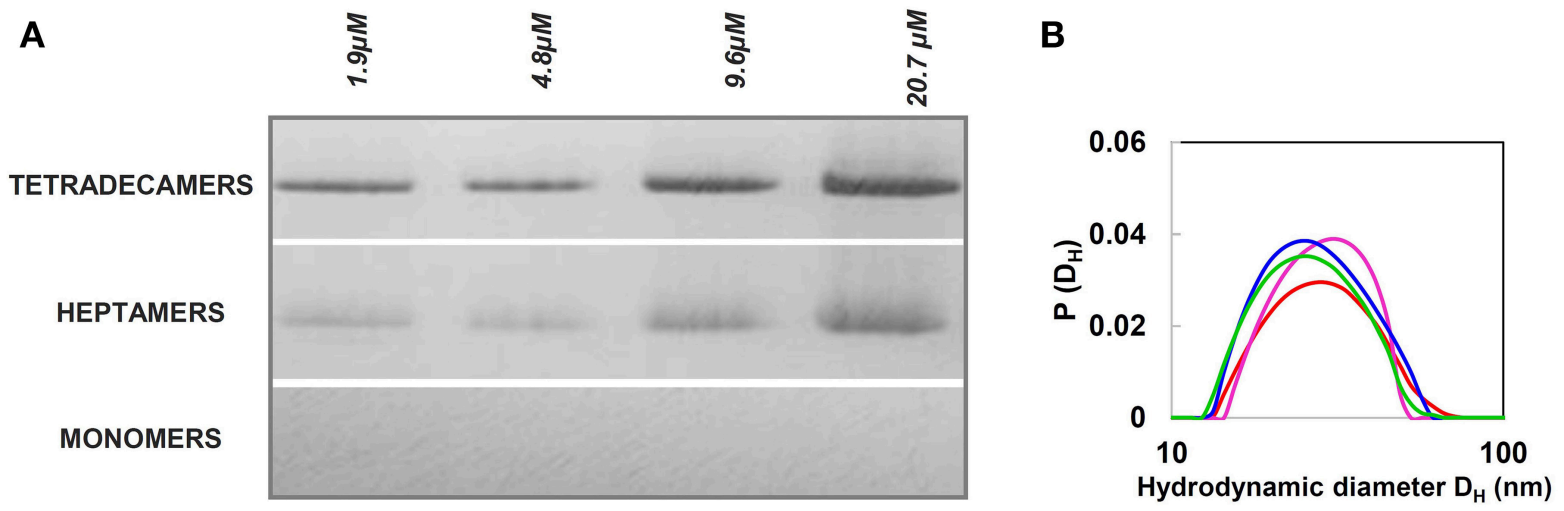
Naïve HSP60 forms oligomers over a wide range of concentrations. **(A)** Blue Native Gel Electrophoresis (4–16%) image of naïve HSP60 in the concentration range 1.9–4.8 μM. The pattern reveals that the protein exists in two oligomeric forms independently of the concentration. **(B)** Size distribution from Dynamic Light Scattering of the naïve HSP60 at various concentrations (1.9 μM: red, 4.8 μM: green, 9.6 μM: blue, 20.7 μM: pink). Adapted from Vilasi et al. (2014). PLOS ONE, according to Creative Commons Attribution (CC BY) policy.

In our studies, we focused on stability and structure of the HSP60 with MTS, which we designate naïve HSP60, as it occurs after being synthesized in the cytosol and before entering the mitochondria (Vilasi et al., 2014). These studies were stimulated by the increased awareness of the potential roles of Hsp60 in cell compartments other than mitochondria and outside the organelle (Soltys and Gupta, 1998; Cechetto et al., 2000; Cappello et al., 2008, 2014; Wick et al., 2014; Wick, 2016; Rahman et al., 2017; van Eden et al., 2017; Calderwood, 2018; Pockley and Henderson, 2018; see other references at http://hsp60.com/localization/). By Electron Microscopy, it has been demonstrated that in normal conditions, 15–20% of Hsp60 is located at extra-mitochondrial sites, namely, in the mitochondrial outer membrane, cytosolic vesicles plasma membrane, endoplasmic reticulum, and peroxisomes (Soltys and Gupta, 1998). These locations seem to correspond to specific physiological functions. As an example, in mature insulin secretory vesicles of pancreatic beta-cells, Hsp60, according to its canonical function, could have a role in insulin core condensation necessary for the hormone release (Soltys and Gupta, 1998). Hsp60 is also involved in assembly of membrane proteins and in the condensation of urate oxidase crystalline core of rat liver peroxisomes. It is especially in pathologic situations such as cancer and autoimmune and inflammatory diseases that HSP60 accumulates in the cytosol, and in extramitochondrial sites (Czarnecka et al., 2006; Desmetz et al., 2008; Cappello et al., 2011, 2014; Macario et al., 2013; Wick et al., 2014; Macario and Conway de Macario, 2016; Rahman et al., 2017; van Eden et al., 2017; Calderwood, 2018; Pockley and Henderson, 2018). The question still open is how the protein reaches these sites, and, consequentially, which forms of Hsp60 can be found in the various locations. One hypothesis is that cytosolic accumulation of Hsp60 could occur via a mitochondrial export mechanism, which would release Hsp60 devoid of MTS into the cytosol (Soltys and Gupta, 1998). In this regard, several possibilities can be considered, such as reverse operation of the mitochondrial import channel, or an as yet undefined export pathway, or a movement through lipids, or an export mechanism involving vesicles. Also, in some cases, Hsp60 can reside and accumulate in the cytosol without being imported into mitochondria and, therefore, this Hsp60 would bear MTS. An example is provided by the LNCaP cells, in which exposure to apoptosis inducers, such as serum starvation or Dox treatment, causes HSP60 accumulation in the absence of mitochondrial release (Chandra et al., 2007). Moreover, an antibody against the MTS crossreacted with a protein that is present only in the cytoplasm of rat liver cell (Itoh et al., 2002) and, although not explicitly commented in the original article, two bands are present in the Western blot (with anti-Hsp60 monoclonal antibody) of the cytoplasmic fraction from adult cardiac myocytes (Kirchhoff et al., 2002). It has been demonstrated that in some apoptotic systems mtHsp60 directly interacts with procaspase-3 in the cytosol, enhancing caspase-3 maturation and activation as part of a pro-apoptotic mechanism (Samali et al., 1999; Xanthoudakis et al., 1999; Chandra et al., 2007). In other systems characterized by naïve Hsp60 accumulation in the cytosol, an anti-apoptotic, pro-survival role is believed to occur, since Hsp60 knockdown or inhibition causes cell death (Kirchhoff et al., 2002; Chandra et al., 2007; Caruso Bavisotto et al., 2017b). In this case, Hsp60 binds procaspase-3 and thereby blocks the apoptotic cascade. In summary, mature mtHsp60 would activate pro-apoptotic mechanisms and, thus, in certain types of cancer it would interfere with cancer cell growth, whereas naïve Hsp60 would be anti-apoptotic and ensure survival of cancer cells. Moreover, it was found that from the cytosol, HSP60 was released by tumor cells via exosomes into the extracellular space (Merendino et al., 2010; Campanella et al., 2012). In this case, by specific immunoprecipitation experiments, two HSP60 forms were found, suggesting the coexistence of mature and naïve forms of the chaperonin. In conclusion, either one or the other, or both forms of HSP60, naïve and mitochondrial, may potentially occur at the various locations in which the chaperonin has been detected in pathological conditions, and this is an issue deserving more investigation. Also, elucidation of the oligomeric organization of the chaperonin in all those locations in which it has been detected in pathological situations will no doubt provide new insights into the molecular basis of disease. For instance, it has been demonstrated that a single mutation in HSP60 found in MitCHAP-60 destabilizes the chaperonin oligomers (Parnas et al., 2009). This is why in view of the potentially key roles of naïve HSP60 in cancer and other diseases, several studies have focused on its oligomeric organization and structure (Vilasi et al., 2014; Ricci et al., 2016, 2017; Enriquez et al., 2017). A detailed study of the structure and self-organization of naïve cytosolic recombinant His-tag HSP60 in solution was performed, using biophysical methods such as Light and X-Ray Scattering, Single Molecule Spectroscopy, and hydrodynamics measurements (Vilasi et al., 2014). *In vitro* experiments were carried out under conditions approximating as much as possible those occurring *in vivo*, using HSP60 at a range of concentrations encompassing those believed to occur *in vivo*: from 10 nM to 79.5 μM. In *E. coli*, the GroEL concentration is estimated to be 35 μM of monomers (Lorimer, 1996). Even if there are no data from direct measurements on the Hsp60 concentrations in the eukaryotic-cell cytosol or mitochondria, we can assume that the range of concentrations described above should include those found *in vivo*, in all cell compartments, under physiological, and stress conditions. Our results showed that HSP60 oligomerizes at all the concentration tested, forming both tetradecamers and heptamers with a prevalence of the former and no detectable monomers (Vilasi et al., 2014). This is illustrated by data from Native Gel Electrophoresis and Dynamic Light Scattering, Figure 5. HSP60 at higher concentrations was analyzed by Small Angle X-Ray Scattering and, at lower concentrations was analyzed by Fluorescence Correlation Spectroscopy (Vilasi et al., 2014). In another laboratory, HSP60 was purified applying a protocol that yields HSP60 without the His6-tag (Enriquez et al., 2017). The results were similar to those described in the preceding lines, although a more marked difference between tetradecamer and heptamer concentrations was found. In this case, the HSP60-His6-tag purification via Affinity Column Chromatography yielded a protein unable to oligomerize and occurring exclusively as monomers. Instead, the protein without His6-tag, studied by EM, Native Gel Electrophoresis, and Light Scattering appeared as tetradecamers with a minor part of heptamers. It has not yet been established what form, monomer, or single or double ring occurs in the cytosol. However, based on the findings described above, we can conclude that naïve HSP60 has biochemical features like those of the mitochondrial mature HSP60 and monomers associate to form the classical toroidal form. We hypothesize that this form does not disassemble before entering, as monomer, into mitochondria. It is likely that cytosolic chaperones, such as Hsp70 and Hsp90, bind the Hsp60 precursor and usher it into mitochondria thus preventing oligomerization in the cytosol. However, we can only say at this time, that in the case of HSP60 accumulation resulting from the failure of the chaperonin precursor to enter into mitochondria, the protein has the ability to form the toroidal structure, probably as necessary to perform its functions in those specific conditions. Future studies should also focus on determining ATPase and in vitro folding activities of naïve HSP60 to establish the extent of its functionality in comparison with the mature molecule.

**Figure 6:**
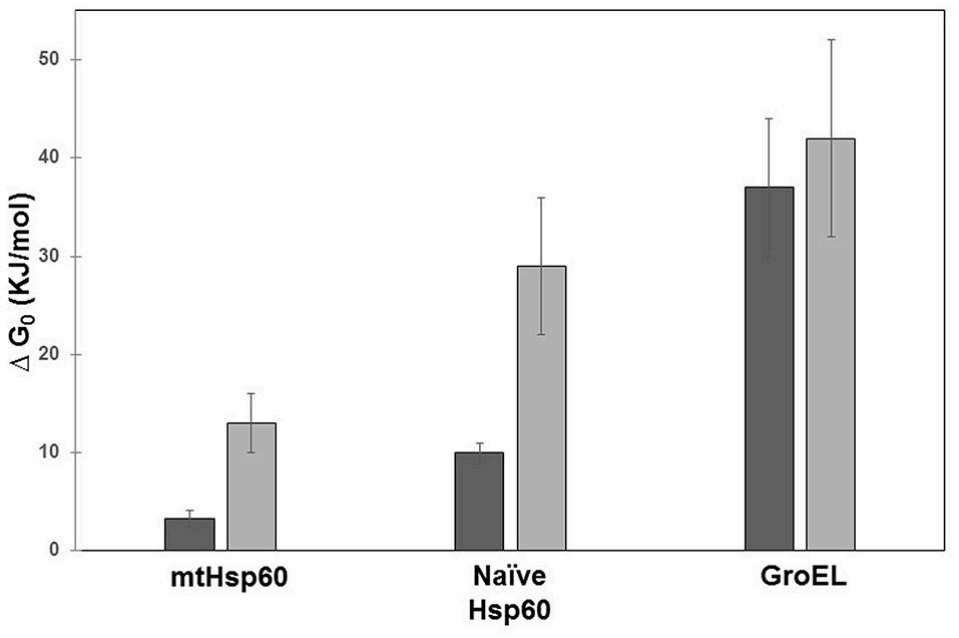
Free energy of unfolding of mitochondrial HSP60, naïve HSP60, and GroEL. The free energy was calculated from the denaturation profile induced by guanidine hydrochloride evaluated by Circular Dichroism (CD) (dark gray) and Small X-Ray Angle Scattering (SAXS) (light gray). Details on data analysis are reported in Ricci et al. (2016, 2017). GroEL appears more stable than human chaperonins and mitochondrial mature HSP60 less stable than naïve HSP60. In contrast to GroEL, significant is the difference between results from the two techniques for the two HSP60 forms. This is due to: a) the different protein concentrations required by the two methods used (orders of magnitude ≈ 1 μM for CD and ≈ 20 μM for SAXS), which have a more marked effect on human chaperonins; and b) the data-analysis model applied, which in the case of CD considers the two-state unfolding model that has been shown to work well with GroEL.

It is worth noting that mature HSP60 produced without His6-tag occurs predominantly as single rings (Nielsen and Cowan, 1998; Parnas et al., 2009), whereas for naïve HSP60 the equilibrium seems to shift toward double rings. Structural differences between the two HSP60 forms can also be inferred from the studies on the chemical stability of the two proteins, (Figure 6) (Ricci et al., 2016, 2017). In order to corroborate this suggestion, further studies are needed to directly compare the two proteins, possibly purified under the same conditions, also in relation to their different activity.

## Hsp60 post-translational modifications

The functions of Hsp60 are closely related to its structure and, therefore, any changes in composition and conformation due to mutations or aberrant post translational modification (PTM) may cause a chaperonopathy.

Most likely, some PTMs occur during the synthesis of the Hsp60 peptide at or near the ribosome and also later, but before the folding process necessary to yield a mature, functional Hsp60 monomer, ready to display its functions alone or as part of a heptamer or tetradecamer. Several lines of research are currently underway aimed to: (a) clarify how PTM change Hsp60 properties and functions and, thereby, its physiological roles; and (b) determine its etiopathogenic activity in chaperonopathies. For instance, a recent study examined HSP60 hyperacetylation during anticancer-drug treatment in human osteosarcoma cells (Gorska et al., 2013). The results lead to the working hypothesis that the post-translational hyperacetylation of HSP60 associated with administration of geldanamycin, contributes to the death of cancer cells.

**Figure 7:**
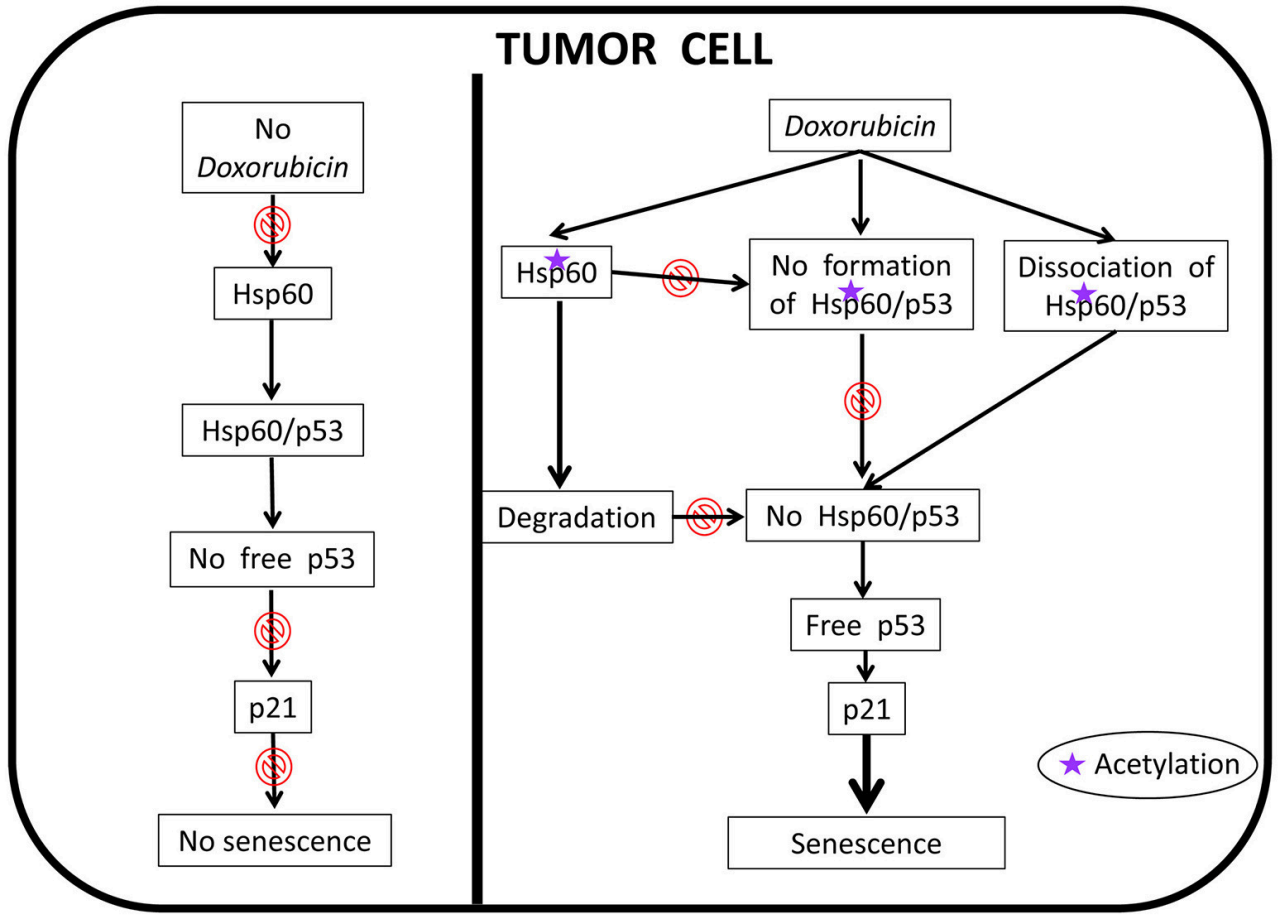
Proposed anti-cancer mechanism of action of doxorubicin involving human HSP60. In the absence of doxorubicin, HSP60 (shown as Hsp60 to encompass not only the human chaperonin but also any other ortholog from animal experimental models) can form complexes with p53 thereby removing free p53 and, thus, there is no interaction between it and p21, which results in the abolition of senescence; the tumor cell is immortal. In this situation HSP60 has an essential pro-tumor effect. Doxorubicin cancels this effect, an action that is associated with HSP60 acetylation. The modified HSP60 is degraded, dissociates from HSP60/p53 complexes, and cannot form de novo these complexes, all of which leads to the occurrence of free p53 that interacts with p21 leading to senescence; the tumor cell now is no longer immortal.

More recently, it was reported that HSP60 hyperacetylation and ubiquitination are associated with the response of cancer cells to administration of the anticancer-drug doxorubicin (Marino Gammazza et al., 2017). Hyperacetylated HSP60 would be directed via ubiquitination to the proteasome system, which would cause a decrease or loss of HSP60 functions, leading to the re-instauration of cellular senescence in cancer cells followed by tumor-cell growth arrest, Figure 7. The HSP60 PTM may lead to disruption of its interaction with other molecules such as p53. These results are in agreement with the observation that HSP60 O-GlcNAcylation impairs its complexing with Bax, leading to cell death (Kim et al., 2006). Also, in line with these data is the finding that HSP60 modifications have an impact on its trafficking, favoring its secretion into the extracellular space (Campanella et al., 2016).

**Figure 8:**
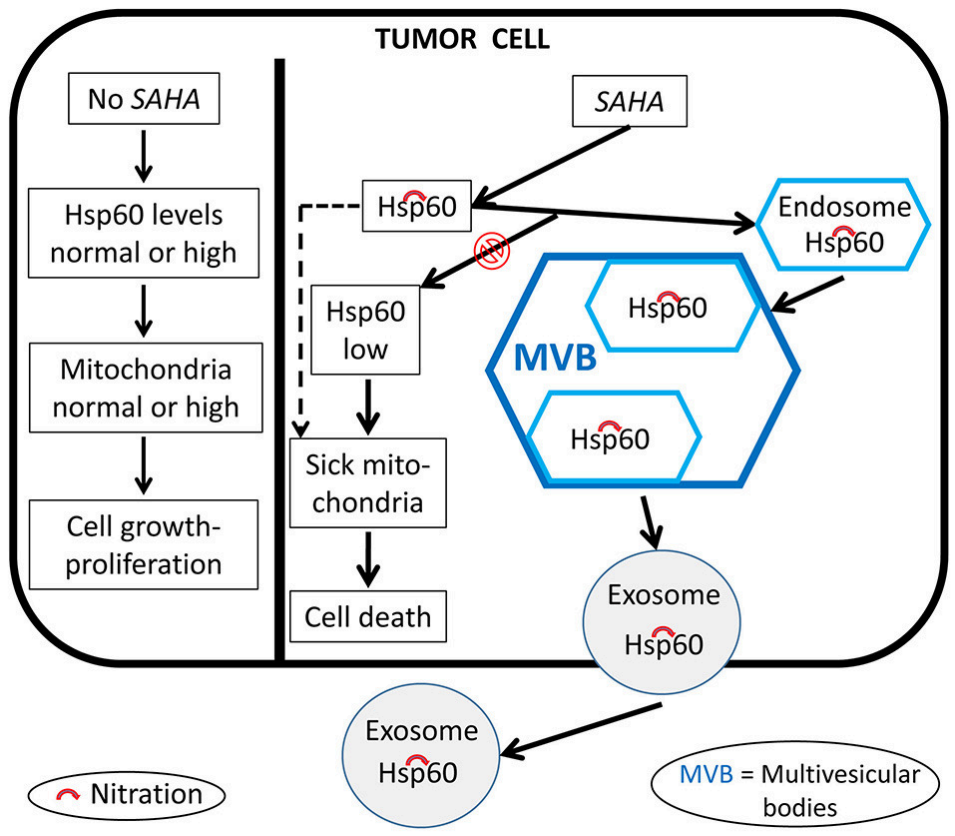
Proposed mechanism of the anti-cancer effects of SAHA involving human HSP60. In the absence of SAHA, the levels and quality of HSP60 (shown as Hsp60 to encompass not only the human chaperonin but also any other ortholog from animal experimental models) and mitochondria are those required by the tumor cell to grow and proliferate. The anti-tumor effect of SAHA modifies these HSP60 parameters. HSP60 levels decrease, the protein is nitrated, i.e., functionally impaired, which leads to sick mitochondria and its capture by endosomes and multivesicular bodies and, ultimately, secretion out of the cell via exosomes; all these events contribute to a decrease of normal HSP60 inside the tumor cell and to impairment of mitochondrial physiology, leading to tumor-cell death.

It was found that the histone deacetylase inhibitor, suberoylanilide hydroxamic acid (SAHA) is cytotoxic for tumor cells, an effect associated with changes in the levels of concentration and nitration of HSP60, Figure 8 (Campanella et al., 2016). The nitrated protein could be exported via extracellular vesicles, such as exosomes (Campanella et al., 2016; Caruso Bavisotto et al., 2017a). Since exosomes are extracellular vehicles that transport factors associated with cancer progression and factors that can modulate the immune response, the presence of HSP60 in them suggests involvement of this chaperonin in inflammation, immune system modulation, and regulation of tumor microenvironment and growth.

In summary, our most recent work has provided new insights, supporting the idea that post-translational modification of HSP60 are associated with key changes inside and outside cells. For instance (i) acetylation is accompanied by a decrease of HSP60 levels and functions such as interaction with p53, and re-instauration of senescence in tumor cells; (ii) acetylation and ubiquitination most likely leads to HSP60 degradation in the proteasome; and (iii) HSP60 nitration affects its trafficking, favoring its translocation into exosomes and subsequent secretion into the circulation, a situation that allows HSP60 to reach target cells, near or far, and thus exercise, for instance, a regulatory action on the immune system. Working hypotheses for studying the effect of PTM on Hsp60 in tumor cells treated with anti-cancer drugs are depicted in Figures 7, 8.

## Conclusions and perspectives

Hsp60 monomers in solution tend to associate into oligomers and form heptameric rings, and double rings, i.e., tetradecamers. The latter seem to be the preferred functional complex. However, single rings may also have functions *in vivo*, without the need for tetradecamer intermediates, under normal and pathologcal conditions. This point deserves more research because single rings may participate in cellular mechanisms which, if elucidated, will enhance our understanding of molecular chaperones and their roles in health and disease. Monomers as such, despite their tendency to oligomerize when in solution, may also play important roles in health and disease. We know that Hsp60 occurs in mitochondria, cytosol, other organelles, the plasma cell membrane, the intercellular space, and in circulation free or attached to corpuscular bodies such as red and white cells, or in exosomes. It is likely that at least in some of these locations Hsp60 is present as monomers, the functions of which deserve active investigation, as much as heptamers and tetradecamers do.

Other structural details of Hsp60 in health and disease that deserve close scrutiny are PTM. These molecular modifications may be crucial for determining: (i) with which of the various possible interactive partners Hsp60 will interact; (ii) the locale in which it will reside and function; (iii); which of the several physiological roles the Hsp60 molecule is able to play will in fact be played; and (iv) whether the chaperonin will be cytoprotective or pathogenic.

## Author contributions

All authors listed have made a substantial, direct and intellectual contribution to the work, and approved it for publication.

### Conflict of interest statement

The authors declare that the research was conducted in the absence of any commercial or financial relationships that could be construed as a potential conflict of interest.
